# A controlled study on the efficacy and quality of life of laparoscopic intersphincteric resection (ISR) and extralevator abdominoperineal resection (ELAPE) in the treatment of extremely low rectal cancer

**DOI:** 10.1097/MD.0000000000020245

**Published:** 2020-05-29

**Authors:** Wenming Yang, Libin Huang, Peng Chen, Yun Yang, Xueting Liu, Cun Wang, Yongyang Yu, Lie Yang, Ziqiang Wang, Zongguang Zhou

**Affiliations:** a Department of Gastrointestinal Surgery, West China Hospital of Sichuan University; b Institute of Digestive Surgery, State Key Laboratory of Biotherapy and Cancer Center, West China Hospital of Sichuan University; c Department of Evidence-Based Medicine and Clinical Epidemiology, West China Hospital of Sichuan University, No. 37 Guoxue Lane, Chengdu; d Department of General Surgery, West China-Ziyang Hospital of Sichuan University/The First People's Hospital of Ziyang, No. 66 Rende West Road, Ziyang; e Department of General Surgery, West China-Shangjin Hospital of Sichuan University/Chengdu Shangjin Nanfu Hospital, No. 253 Shangjin Road, Chengdu, China.

**Keywords:** laparoscopic extralevator abdominoperineal excision, laparoscopic intersphincteric resection, lower rectal cancer, neoadjuvant chemoradiation, quality of life, survival outcome

## Abstract

**Background:**

The aim of this study is to compare the postoperative quality of life (QoL) and survival outcomes in lower rectal cancer (LRC) patients who undergo either laparoscopic- intersphincteric resection or extralevator abdominoperineal excision (L-ELAPE) after long-course neoadjuvant chemoradiation therapy (nCRT).

**Methods:**

This prospective, single-center, non-randomized, controlled, non-blinded, phase I/II clinical trial is designed to enroll 159 eligible LRC patients who achieved favorable response to long-course nCRT (2 × 25 Gy). After informed consent, the patients will be assigned into the laparoscopic intersphincteric resection group or L-ELAPE group according to their own will. Standard radical laparoscopic surgeries will be performed for every participant. Then every participant will be followed up for 3 years. The primary outcomes are scores of QoL questionnaire-core 30, QoL questionnaire-colorectum 29, Wexner incontinence score, International Prostate Symptom Score (for male), International Index of Erectile Function-5 (for male) and Female Sexual Function Index (for female). The secondary outcomes consist of incomplete circumferential resection margin rate, 3-year local recurrence, 3-year disease-free survival, 3-year overall survival and other surgical outcomes.

**Discussion:**

This is the first prospective clinical controlled trial to assess postoperative QoL and efficacy for LRC patients after favorable long-course nCRT. The result is expected to provide new evidence for a more detailed individualized treatment guideline for LRC.

**Trial registration:**

This trial was registered at Chinese Clinical Trial Registry (*ChiCTR1800017512*; ChiCTR.org) on August 2, 2018.

## 1 Introduction

Colorectal cancer is the fourth most commonly diagnosed malignancy and the second predominant cause of cancer-related death worldwide.^[1]^ According to the data from the International Agency for Research on Cancer (IARC), rectal cancer would rank 8th in terms of incidence, accounting for approximately 40% of Colorectal cancer and constituting a severe global public health burden. Surgery is still the cornerstone of curative intent treatment for rectal cancer.^[2]^ Without regard to lower rectal cancer (LRC), it is undoubtful that anterior resection (AR) is the preferred surgical procedure for mid-upper rectal cancer.^[3]^ Abdominoperineal excision (APE) firstly reported by Ernest Miles in 1908 has long been considered the “gold standard” surgical management for LRC.^[4,5]^ Proven unappealing oncologic outcomes in APE compared to AR from several large-scale case series on resected rectal specimen, Holm et al presented a new approach of extended APE, also known as extralevator APE (ELAPE), without waist to reduce the risks of circumferential resection margin (CRM) involvement and intraoperative perforation for advanced tumors.^[6–8]^ Since Schiessel et al proposed their initial experience with intersphincteric resection (ISR) for LRC,^[9]^ a promising alternative to APE without permanent colostomy, the indication for ISR has been gradually assessed and strictly applied. Meanwhile, laparoscopic-assisted minimally invasive surgery is expected to allow meticulous precise operation with good visualization in narrow pelvic cavity.^[10]^

However, with the widespread adoption of neoadjuvant chemoradiation therapy (nCRT), management of LRC has shifted significantly.^[11,12]^ Yet, whether ultimate sphincter-preserving procedures, such as ISR, offer equal or better oncologic outcomes to LRC patients after favorable response from nCRT becomes controversial compared to APE or ELAPE.^[13,14]^ Surgery for LRC has to maintain a balance between radical tumor removal to minimize the risk of locoregional recurrence and patients’ desire, or postoperative quality of life (QoL).^[15,16]^ Postoperative continence is one of important outcome measures of QoL for LRC patients, and there are conflicting reports on the functional outcomes and QoL for patients after ISR compared to APE.^[16–19]^

Thus, the aim of our trial is to prospectively compare QoL, perioperative complications and oncologic outcomes of laparoscopic intersphincteric resection (L-ISR) with laparoscopic extralevator abdominoperineal excision (L-ELAPE) in the patients with LRC after significant response to nCRT. The results are expected to provide new evidences for more detailed individualized therapies for LRC.

## 2 Methods

### 2.1 Study design

**Figure 1:**
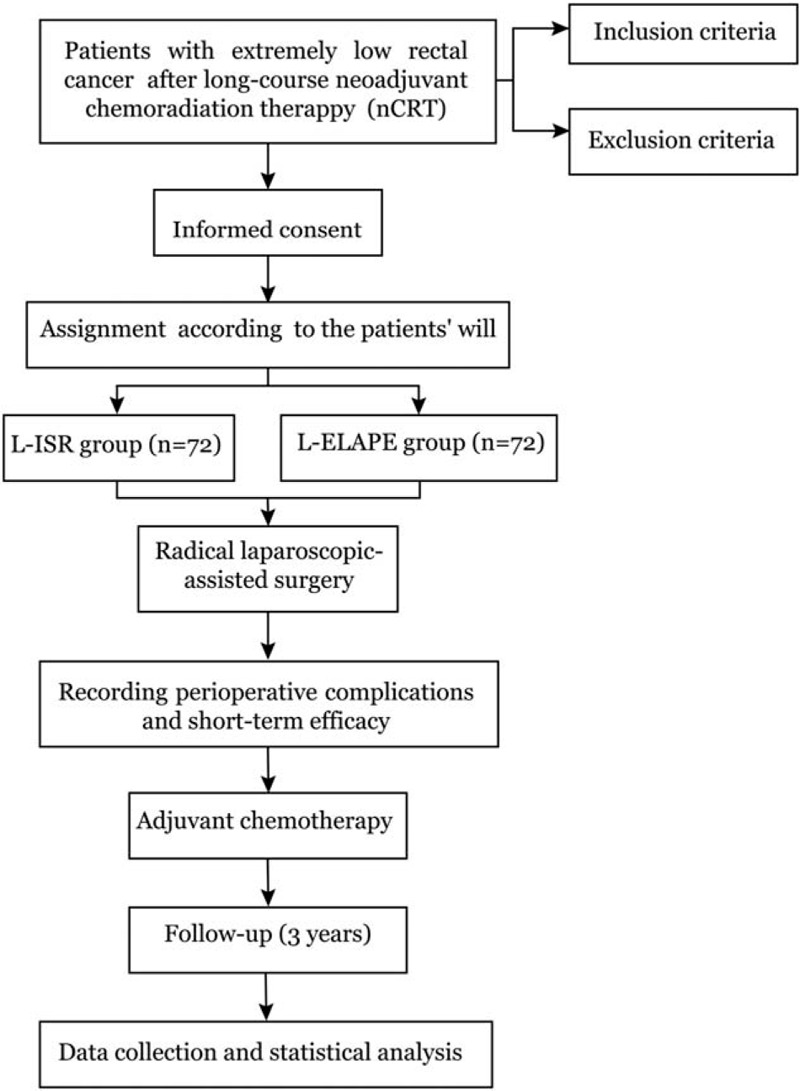
Flow diagram for the schedule of enrollment, interventions, and assessments. L-ISR = laparoscopic intersphincteric resection, L-ELAPE = laparoscopic extralevator abdominoperineal excision.

The study protocol follows the standard protocol items: recommendation for interventional trials (SPIRIT) guidelines.^[20]^ This is a prospective, single-central, non-randomized, non-blinded, phase I/II clinical controlled trial. All eligible patients will be assigned into 2 comparison groups (the L-ISR group and the L-ELAPE group) according to their own will after detailed preoperative conversations. The flow diagram of this trial is shown in Figure 1. Our trial started from September 2018 in West China Hospital of Sichuan University and is expected to end in 2021.

### 2.2 Study population

#### 2.2.1 Inclusion criteria

- Age(years): 18 to 75
- Histologically confirmed rectal adenocarcinoma, such as well/moderately/poorly differentiated tubular, papillary, or mucinous adenocarcinoma, or signet-ring cell carcinoma, etc
- Lower tumor margin is ≤5 cm from anal verge when diagnosed via colonoscopy
- Clinical staging: cT_1–2_N_1–2_M_0_ or cT_3_N_0–2_M_0_ on high resolution magnetic resonance imaging (MRI) before nCRT (according to the 8th edition of AJCC/TNM staging system)
- Six to 12 weeks after long-course nCRT (2 × 25 Gy)
- Absence of multiple primary carcinomas or distant metastasis
- ASA (American Society of Anesthesiologists) classification: I-III
- Performance Status (Eastern Cooperative Oncology Group, ECOG) scores: 0 to 2
- Written informed consent.

#### 2.2.2 Exclusion criteria

- Age(years): younger than 18 or older than 75
- Past history of malignancy
- Rectal cancer related complications (including acute obstruction, perforation, and bleeding) that requiring emergency operations
- Past history of colorectal surgeries which may have impact on the reconstruction of digestive tract this time
- Invasion or suspicious invasion of tumor into the adjacent structure, such as vagina, prostate, sacrococcyx, or lateral pelvic wall
- ASA (American Society of Anesthesiologists) classification: IV-V
- Pregnant or lactating women
- Severe psychological disorder
- Severe emphysema, pulmonary fibrosis, ischemic heart disease (IHD), or other medically inoperable
- Continuous systematic steroid therapy within the past month
- Contraindications of laparoscopic surgery, MRI or radiation therapy
- Patients who cannot understand the requirements and aims of this trial.

### 2.3 Interventions and follow-up

#### 2.3.1 Interventions

After assignment, patients in the L-ISR group will undergo standard L-ISR surgeries, while patients in the L-ELAPE group will undergo standard L-ELAPE surgeries with no intraoperative position change. Prophylactic ileostomy will be performed for selected patients with higher risk of anastomotic leakage in the L-ISR group. The radical laparoscopic-assisted surgery with meticulous total mesorectal excision and pelvic automatic nerve preservation (PANP)^[21–23]^ will be performed independently by 2 experienced and highly-specialized surgeons from the Department of Gastrointestinal Surgery, West China Hospital. Adjuvant chemotherapy, consistent with the preoperative neoadjuvant chemotherapy strategy for every participant, will continue within 4 to 6 weeks following surgery till completing 8 cycles. Closure of the ileostomy will be performed within 3 to 6 months after L-ISR according to rigorous preoperative assessment by digital rectal examination, colonoscopy, and barium enema examination.

#### 2.3.2 Follow-up and dropping out

All participants will be followed up regularly as per schedule (Table 1) every 3 months for the first 3 years after surgeries. If necessary, biopsy and MRI will be performed to assess local or distant recurrence.

**Table 1:**
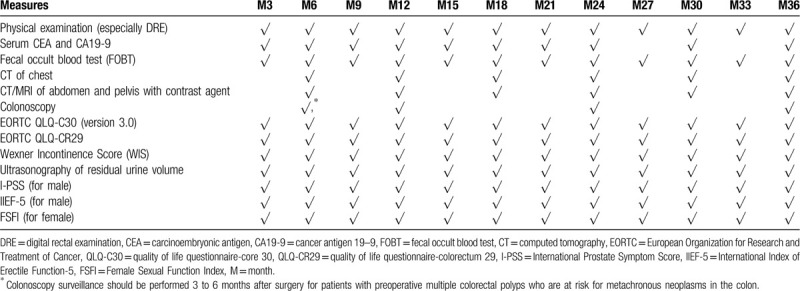
The schedule of follow-up after surgery.

Despite of written informed consent, patients are aware that they are free to discontinue their participation any time after it begins if:

1) the participant is not willing to continue on his or her own for any reason;
2) adverse events which cannot be tolerated occur;
3) the investigators remove a participant off for his or her good;
4) the participant acquired some other treatment privately which will disturb the observation of this trial;
5) pregnance happens to the participant during this trial, or
6) the participant's adherence is poor.

### 2.4 Outcome measures

The primary outcomes of this trial are to be scores of European Organization for Research and Treatment of Cancer QoL questionnaire-core 30 (QLQ-C30), QoL questionnaire-colorectum 29 (QLQ-CR29), Wexner Incontinence Score, International Prostate Symptom Score (for male), International Index of Erectile Function-5 (for male) and Female Sexual Function Index (for female). General QoL is assessed basically by scores of European Organization for Research and Treatment of Cancer QLQ-C30 version 3.0^[24]^ and QLQ-CR29.^[25]^ Defecation functions (daily defecation, frequency, urgency, incontinence, differentiation between defecation and gas emission, pad use, antidiarrheal drug use, and the presence or absence of erosion around the anus) are evaluated by the Wexner Incontinence Score.^[26]^ Sexual function for men and women is assessed by using the International Index of Erectile Function-5^[27]^ and Female Sexual Function Index,^[28]^ respectively. Urinary function is assessed by using the International Prostate Symptom Score^[29]^ for men and color ultrasonography of residual urine volume for all.

The secondary outcomes include incomplete CRM rate, 3-year local recurrence, 3-year disease-free survival, 3-year overall survival, and other surgical outcomes. Incomplete CRM is defined as tumor within 1 mm from closest mesorectal fascia, or within 1 mm from closest levator muscle, or invasion into or beyond the insphincteric plane. Local recurrence is defined as recurrent disease in the pelvis or at the incision. Disease-free survival is defined as the time of surgery to recurrence or end of follow-up. Overall survival is defined as the time of surgery to the date of death from any cause or the end of follow-up. Surgical outcomes include operation time, intraoperative blood loss, temporary stoma, intraoperative perforation, time to first flatus, time to liquid diet intake, postoperative hospitalization days, postoperative complications, incidence of urinary retention, and postoperative mortality in 30 days.

### 2.5 Sample size

**Figure 2:**
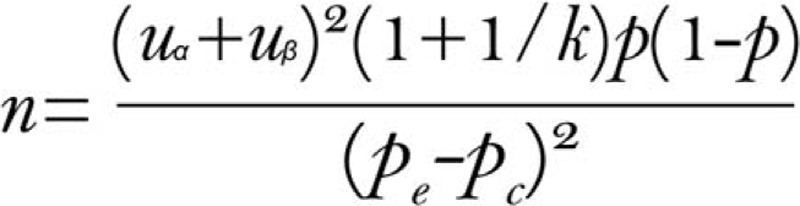
The adjusted formula for sample size calculation. n = sample size, α = probability of type I error, β = probability of type II error, k = the ratio of 2 groups’ sample sizes, p = joint probability, p_e_ = incomplete circumferential resection margin rate of the experiment group, p_c_ = incomplete circumferential resection margin rate of the control group.

This is a non-inferiority verification study with QoL and efficacy as the main index. In the pilot study, incomplete CRM rate was 6.1% (2 out of 33 patients) in the L-ELAPE group. Based on the previous studies, we assume that the rate of incomplete CRM in the L-ISR group was 8.1% which was the average value in the meta-analysis.^[30]^ The adjusted formula below is used to calculate the sample size^[31]^ (Fig. 2). The “Pe” and “Pc” are the rates of incomplete CRM of the experiment group and the control group, respectively. The “k” represents the ratio of 2 groups’ sample sizes which equals 1 in this trial and “p” represents the joint probability. Setting type I error probability **α** = 0.025, type II error probability **β** = 0.2, the sample size required for each group is 72 cases. Furthermore, we assume that there will be a 20% dropout rate. Therefore, at least 159 patients are needed in this specific trial.

### 2.6 Randomization and blinding

Due to the specificity and ethics, it is unlikely that any surgical center around the world would randomize patients to receive either L-ISR or L-ELAPE due to a lack of equipoise regarding the requirement for each operation in any particular patient. Direct comparison by a randomized controlled trial of the 2 procedures is currently virtually impossible. This trial allows the surgeons and outcome assessors to be aware of the whole assignment and all eligible patients will be informed of the treatment in detail on admission to hospital. However, statisticians responsible for the final analyses will be blinded.

### 2.7 Data collection, management, and statistical analyses

For purpose of acquiring high-quality monitoring, we plan to employ several professional clinical investigators to collect and manage the data of all participants during entire trial performing. A Case Report Form (CRF) is designed for each participant to document their data. And we will upload the data of all participants to Research Manager (ResMan), an Electronic Data Capture (EDC) system, and the individual patient data (IPD) will be publicly accessed via Chinese Clinical Trial Registry (ChiCTR.org) within 3 years after completion of this trial.

SPSS for Windows, version 22.0 (IBM Corp, Armonk, NY) will be used for statistical analysis. The data are presented as the mean value **±** standard deviation for continuous variables and as a number for categorical variables. The difference of categorical data and continuous data will be compared with Student *t* test and chi-square test between the 2 groups, respectively. The Kaplan–Meier estimate will be used to analyze survival and fit survival curves. The multiple factor analysis will be performed by using Cox regression model.

### 2.8 Monitoring

#### 2.8.1 Safety and adverse effects

Conventionally, we will carefully and objectively record and report all the adverse events, including perioperative complications and adverse events during follow-up. Furthermore, the severe adverse events will be dealt with in the same way every 6 months and the life-threatening within 24 hours.

#### 2.8.2 Ethics and dissemination

Before enrollment of the first patient, the current trial has been approved by the Clinical Research and Biomedical Ethical Committee (CRBEC) of West China Hospital of Sichuan University (WCH IRB 2018–057; May 17, 2018) and also registered at ChiCTR.org (ChiCTR1800017512; August 2, 2018). Written informed consent will be obtained from every patient for acquisition and utilization of anonymized clinical data prior to recruitment. The purpose and process of the research will be introduced in detail to the eligible patients. Only patients who have understood the purpose and risk of the research and signed the informed consent can participate in this trial. This trial will be conducted in accordance with the study protocol and the principles of the Declaration of Helsinki.^[32]^

The current study is funded by Wu Jieping Medical Foundation. All authors declare that there are no conflicts of interest to disclose. The collected data will be stored for 3 years after termination of this study. The information obtained through the data analyses will be published in an academic journal and publicly shared. Before being completely destroyed, data are available from the responsible investigators upon reasonable request and with permission of Chinese Clinical Trial Registry.

## 3 Discussion

Currently, the hot issue of the treatment for LRC located within 5 cm from anal verge is still being debated worldwide. The rapid improvement of surgical techniques and devices and universal utilization of preoperative treatment brings not only opportunities to management of LRC but new challenges to overcome. There is much controversy, however, on a new question as to which surgical option is more appropriate for patients with LRC after favorable response to nCRT to obtain better postoperative QoL and survival outcomes.

Although recent reports suggest that ultimate sphincter-preserving procedures, including ISR, may be feasible to cure LRC without compromise of resection margins and subsequent survival outcomes,^[13,33]^ advanced surgical technique is indispensable for ISR to deliver oncologic efficacy. There is a steep learning curve to successfully completing the L-ISR or L-ELAPE operation, including precise knowledge of rectal anatomy and its anomalies, to avert injury to critical adjacent structures. Perineal wound complications will be attached great importance on and biomaterial as a novel alternative will be used for pelvic floor reconstruction in the L-ELAPE group of our study. As for ISR, anastomotic leakage and anastomotic stricture must be taken into further consideration.^[34,35]^ Thus, prophylactic ileostomy will be performed for selected patients with higher risk of anastomotic leakage and digital rectal examination monthly after surgery is necessary in the L-ISR group.

Given the evident psychological and social impact of permanent colostomy required by APE surgeries, ISR was developed to provide a sphincter-preserving alternative.^[36,37]^ However, recent studies have challenged the assumption that global QoL is worse in patients with permanent stomas (eg, ELAPE patients).^[18]^ A matched-pair analysis comparing the long-term functional outcomes and QoL for 2 post-surgical populations indicated that ISR was associated with higher frequency of diarrhea and constipation than APE.^[16]^ Postoperative continence is exactly an important clinical outcome for LRC patient. Therefore, the pros and cons of each procedure will be discussed with patients and their families before decision making in our study. Psychological guidance, incontinence and stoma care education, and instruction of sphincter exercises will be added into the follow-up for both groups.

To our knowledge, this is the first prospective clinical controlled trial to evaluate postoperative QoL and efficacy for patients with LRC after favorable long-course nCRT. We will conduct the trial with high quality control and publish the result which is expected to provide new evidence for a more detailed individualized treatment guideline for LRC.

## Author contributions

**Conceptualization:** Wenming Yang, Lie Yang, Yongyang Yu.

**Methodology and statistical analysis:** Wenming Yang, Lie Yang, Xueting Liu, Cun Wang, Yongyang Yu.

**Software and data curation:** Libin Huang, Peng Chen, Yun Yang, Xueting Liu, Cun Wang.

**Supervision and validation:** Lie Yang, Yongyang Yu, Ziqiang Wang, Zongguang Zhou.

**Writing – original draft:** Wenming Yang, Libin Huang, Peng Chen.

**Writing – review & editing:** Lie Yang, Yongyang Yu.
